# Supplementary material for: Contribution of irreversible non-180° domain to performance for multiphase coexisted potassium sodium niobate ceramics
Source: Nat Commun. 2024 Mar 18;15:2408. doi: 10.1038/s41467-024-46800-z (PMC10948830; doi:10.1038/s41467-024-46800-z)
Supplement: Supplementary file 1 — Supplementary Information [file 41467_2024_46800_MOESM1_ESM.pdf]

Supplementary Materials

Contribution of irreversible non-180° domain to performance for multiphase coexisted potassium sodium niobate ceramics

Bo Wu[1,2,3], Lin Zhao[1,3], Jiaqing Feng[1,3], Yiting Zhang[1,3], Xilong Song[1,3], Jian Ma[1,3], Hong Tao[1,3]*, Ze Xu[2], Yi-Xuan Liu[2], Shidong Wang[4]*, Jingtong Lu[2], Fangyuan Zhu[5], Bing Han[6]* and Ke Wang[2]*

[1]Sichuan Zoige Alpine Wetland Ecosystem National Observation and Research Station, Southwest Minzu University, Chengdu, 610225, P. R. China.

[2]State Key Laboratory of New Ceramics and Fine Processing, School of Materials Science and Engineering, Tsinghua University, Beijing, 100084, P. R. China.

[3]Sichuan Province Key Laboratory of Information Materials, Southwest Minzu University, Chengdu, 610225, P. R. China.

[4]Musculoskeletal Tumor Center Peking University People's Hospital, Beijing, 100044, P. R. China.

[5]Shanghai Synchrotron Radiation Facility, Shanghai Advanced Research Institute, Chinese Academy of Sciences, Shanghai, 201204, P. R. China.

[6]Department of Orthodontics, Peking University School and Hospital of Stomatology, Beijing, 100081, P. R. China.

*Corresponding author: taohongscu@163.com (Hong Tao); stonewang@bjmu.edu.cn (Shidong Wang) ; kqbinghan@bjmu.edu.cn (Bing Han); wang-ke@tsinghua.edu.cn (Ke Wang)

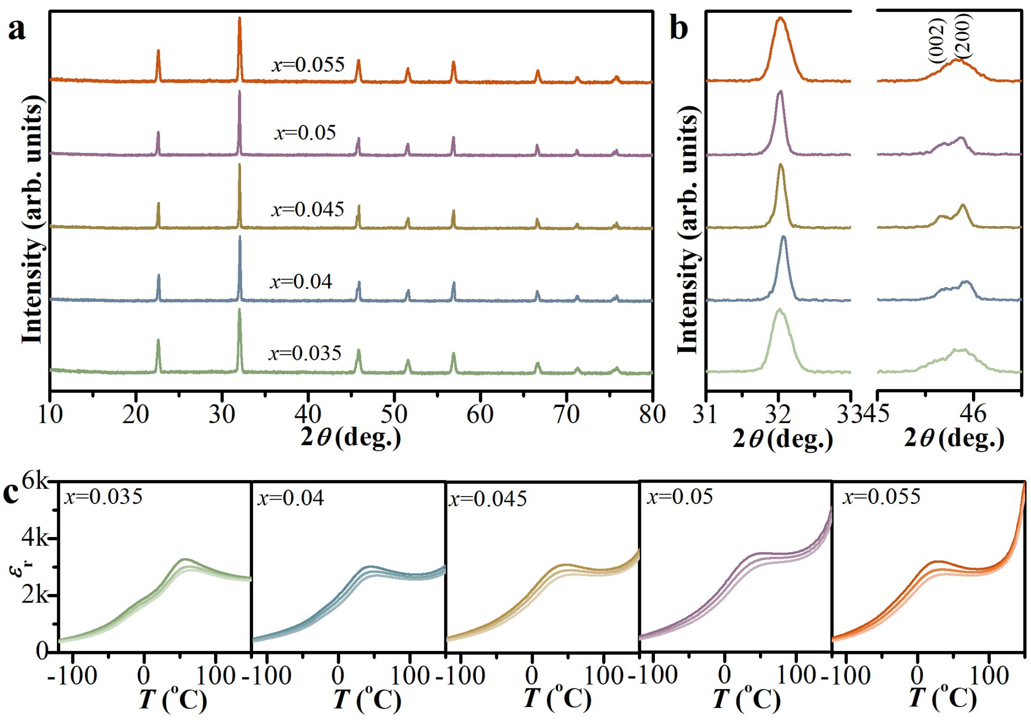

Supplementary Fig. 1 XRD patterns of the ceramics with different x: **a** 2θ=10~80°, **b** 2θ=31-33° and 2θ=45-47°. **c** ε_r-T curves of the ceramics measured at -120~150 °C.

Supplementary Table 1. Phase transition temperature for the ceramics.

x	$T_{R\text{-}O}$ (°C) (approximate)	$T_{O\text{-}T}$ (°C)	T_C (°C)
0.035	-30	64	235
0.04	-26	48	218
0.045	-24	41	196
0.05	-27	42	191
0.055	~	28	178

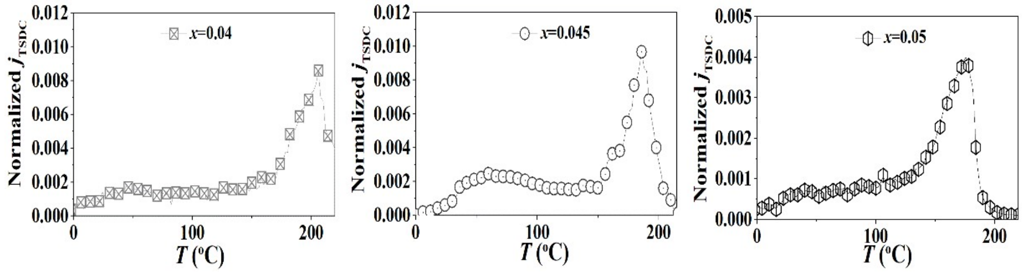

Supplementary Fig. 2 Normalized temperature dependence of depolarization current (j_{TSDC}) of the ceramics.

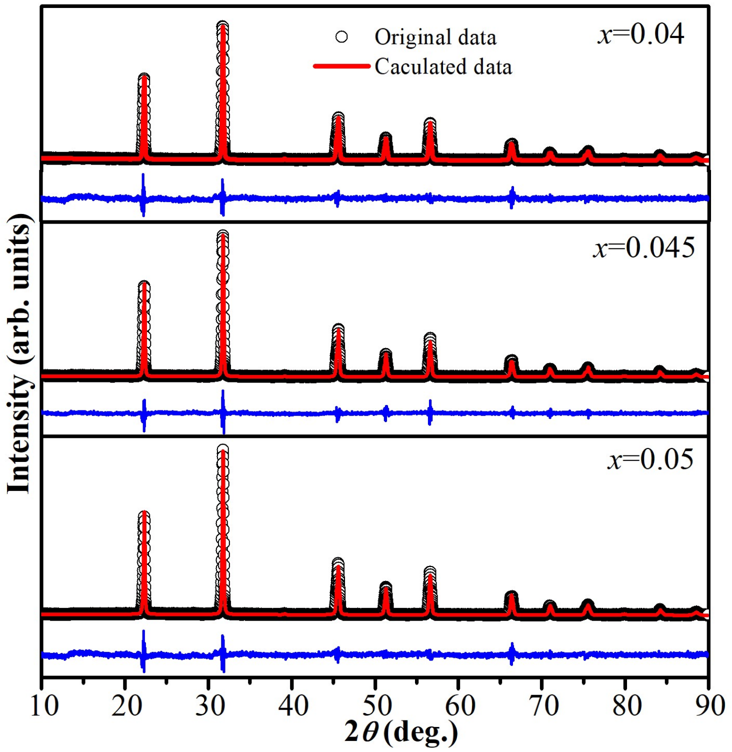

Supplementary Fig. 3 Rietveld refinement of the ceramics.

Supplementary Table 2. Structure parameters for the ceramics with different compositions, measured at 25 ºC.

x	Sig	R_w (%)	Space group	a (Å)	b (Å)	c (Å)	Alpha(º)
			R3m	3.9863	3.9863	3.9863	89.8334
0.04	1.61	2.08	Amm2	3.9777	5.6304	5.6382	-
			P4mm	3.9742	3.9742	3.9966	-
			R3m	3.9858	3.9858	3.9858	89.7745
0.045	1.71	2.09	Amm2	3.9718	5.6365	5.6365	-
			P4mm	3.9740	3.9740	4.0048	-
			R3m	3.9901	3.9901	3.9901	89.9228
0.05	1.73	2.06	Amm2	3.9725	5.6407	5.6484	-
			P4mm	3.9760	3.9760	3.9848	-

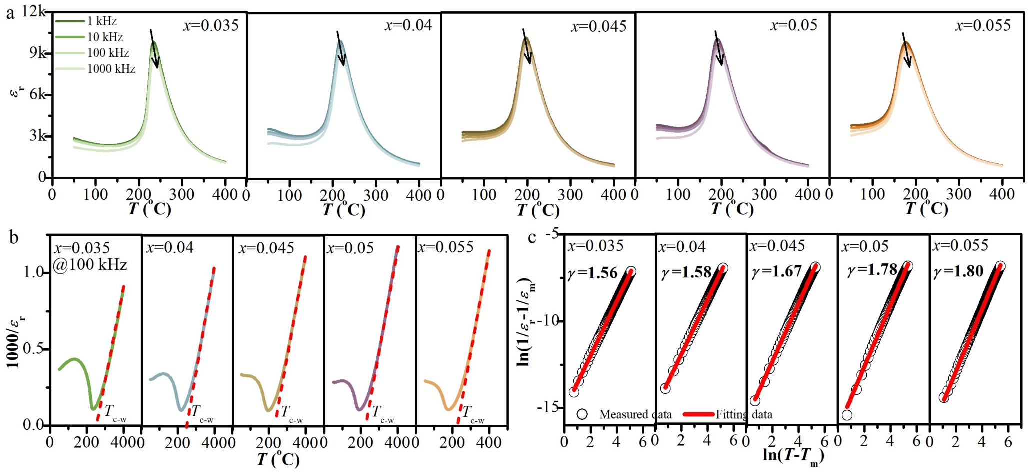

Supplementary Fig. 4. Dielectric behavior of the ceramics. a ε_r-T curves for the ceramics at 50~400 ºC. **b** 1000/ε_r-T curves. **c** ln(1/ε_r-1/ε_m) as a function of ln(T-T_m).

Supplementary Table 3. Electric properties for the ceramics.

x	***d*₃₃ (pC/N)**	***k*p**	***Qm***	***εr***	**tan*δ***
0.04	495	0.482	56	2464	0.028
0.045	530	0.496	54	2749	0.024
0.05	505	0.495	59	2995	0.032

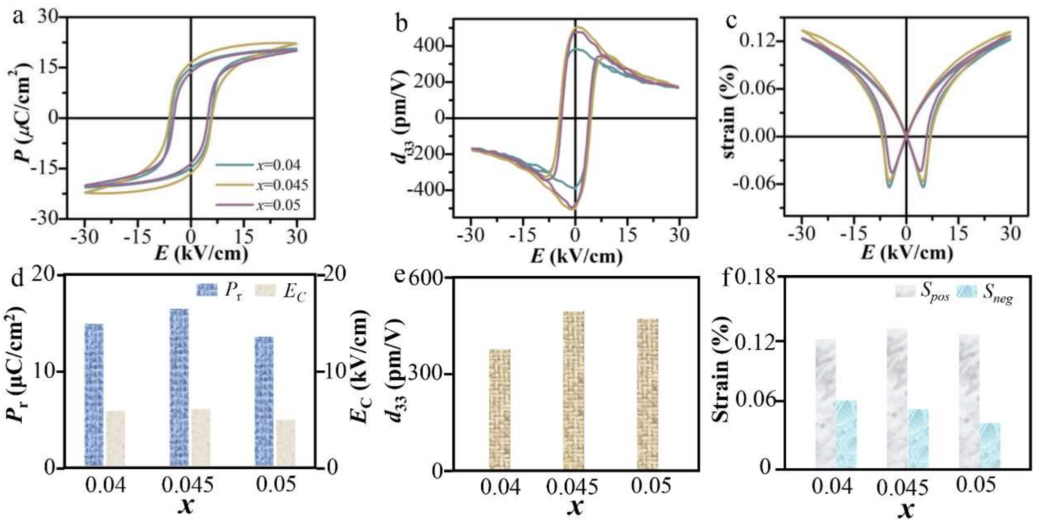

Supplementary Fig. 5. Ferroelectric, piezoelectric, and strain property of the ceramics. a *P-E* loop. **b** Electric field-induced d_{33} curves. **c** Bipolar strain curves. **d** P_r and E_C. **e** d_{33}, **f** S_{pos} and S_{neg} for the ceramics.

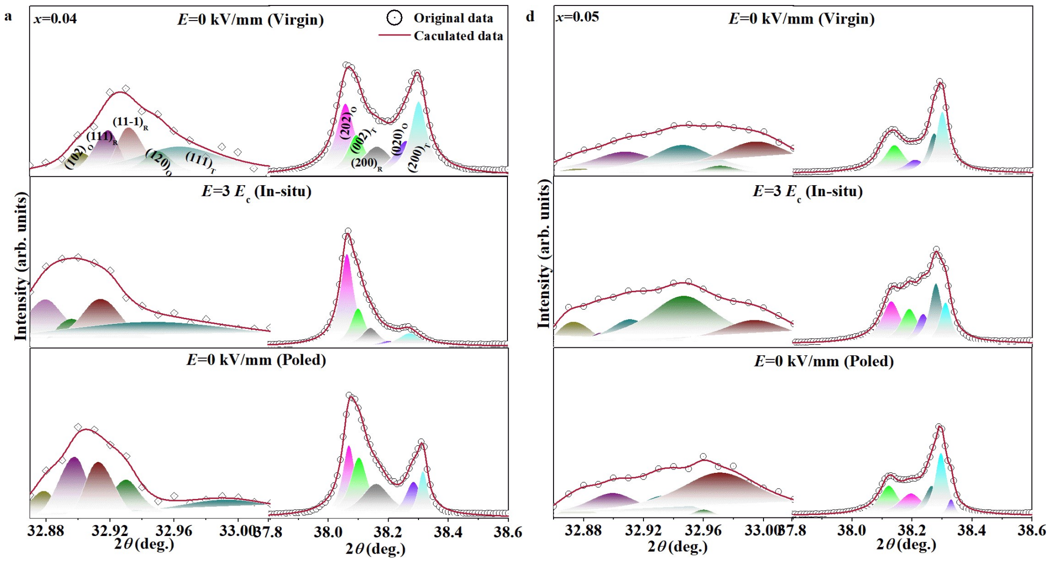

Supplementary Fig. 6 *In-situ* synchrotron XRD patterns of (111) and (200) reflections as a function of the electric field in the ceramics: **a** x=0.04, **b** x=0.05.

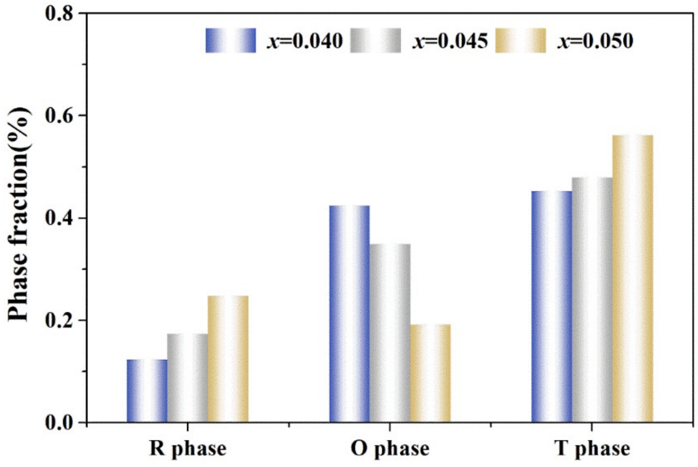

Supplementary Fig. 7 Phase fraction of the ceramics calculated by *in-situ* synchrotron XRD patterns.

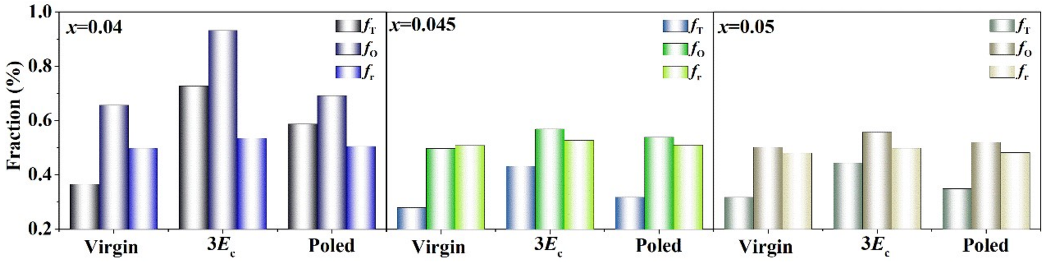

Supplementary Fig. 8 The volume fraction of domains parallel to the electric field direction for R, O, and T phases.

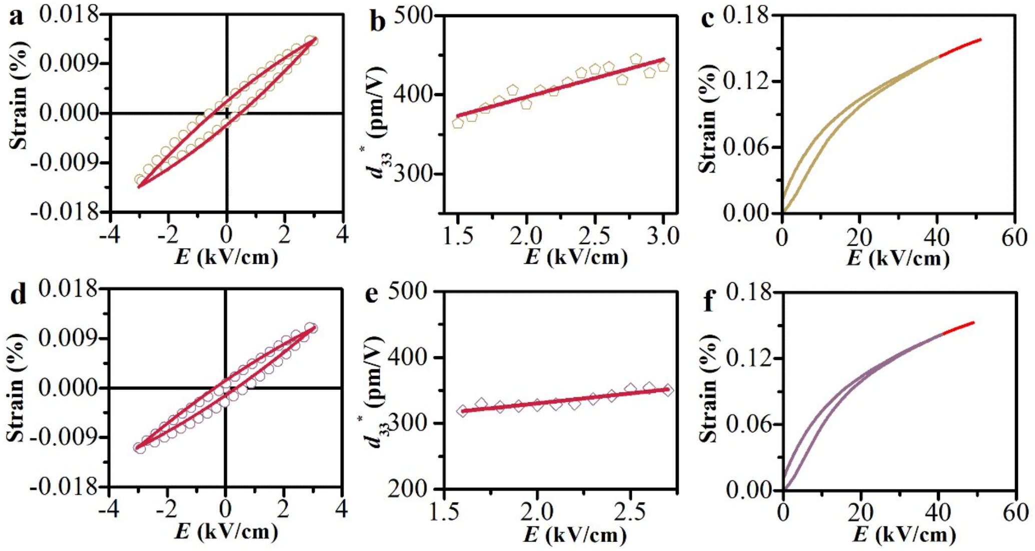

Supplementary Fig. 9 Rayleigh analysis for x=0.04: **a** measured and fitted electric field strain curves, **b** piezoelectric coefficient with respect to the amplitude of electric field, **c** unipolar strain curves at a high electric field. Rayleigh analysis for x=0.05: **d** measured and fitted electric field strain curves, **e** piezoelectric coefficient with respect to the amplitude of electric field, **f** unipolar strain curves at a high electric field.

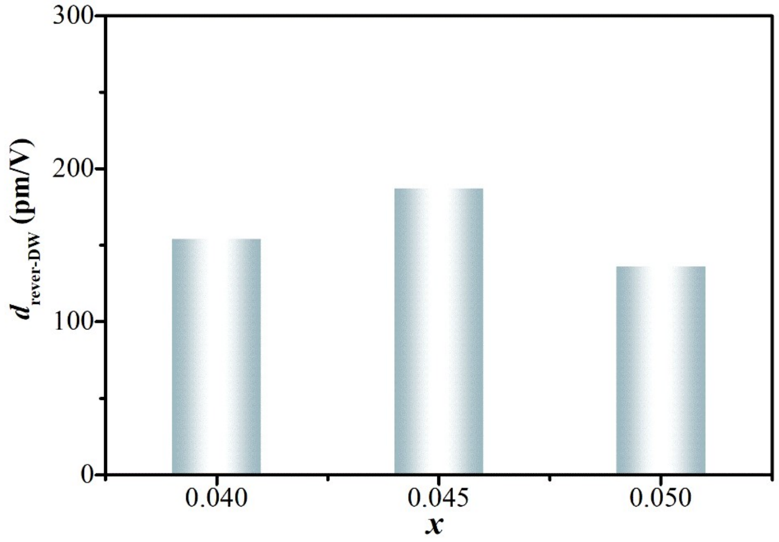

Supplementary Fig. 10 Piezoelectric response from reversible domain wall motion as a function of x.

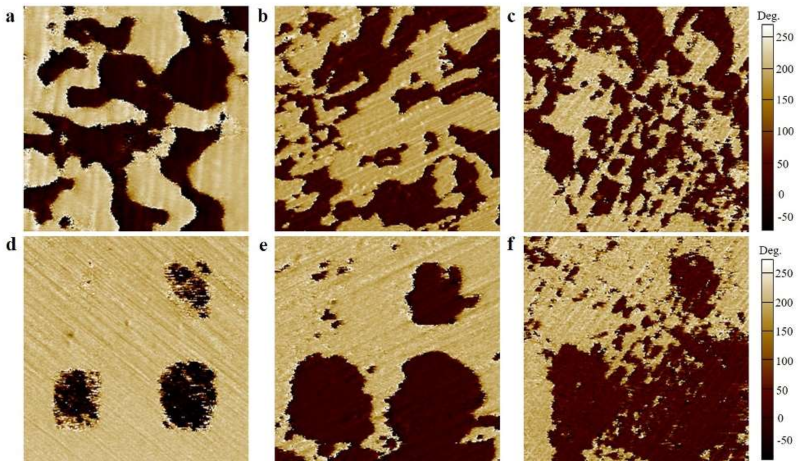

Supplementary Fig. 11 Phase images of the ceramics. Phase image of virgin domain: **a** x=0.04, **b** x=0.045, and **c** x=0.05. Phase images after litho process under bias: **d** x=0.04, **e** x=0.045, and **f** x=0.05.

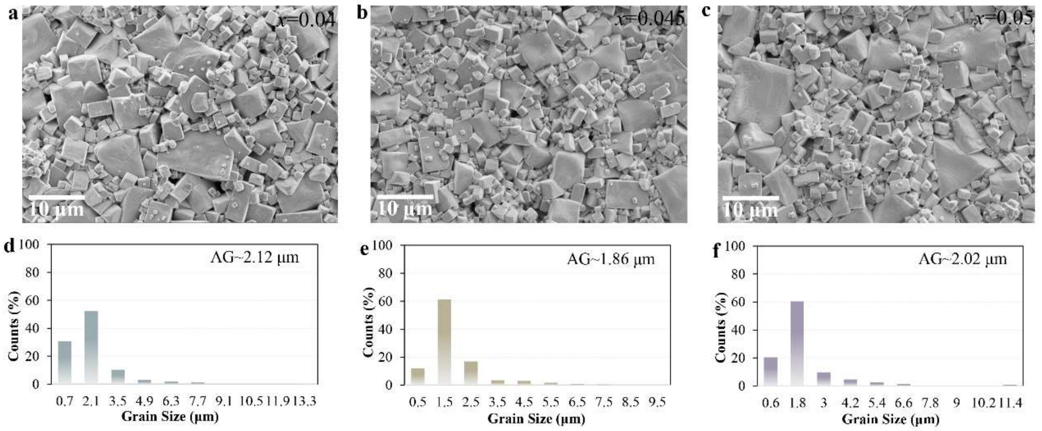

Supplementary Fig. 12 Grain morphology. FE-SEM images of the ceramics: **a** x=0.04, **b** x=0.045, **c** x=0.05; statistics of grain size: **d** x=0.04, **e** x=0.045, **f** x=0.05.

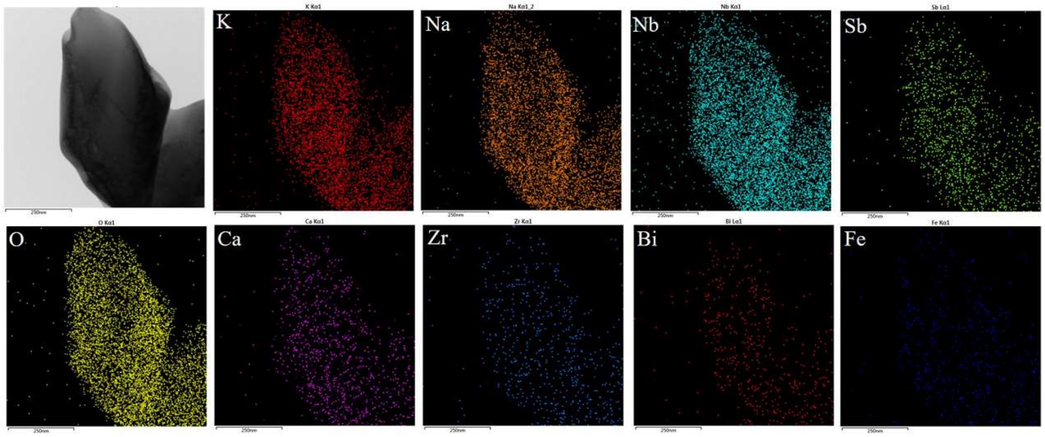

Supplementary Fig. 13 Element mapping of the ceramic with x=0.045.

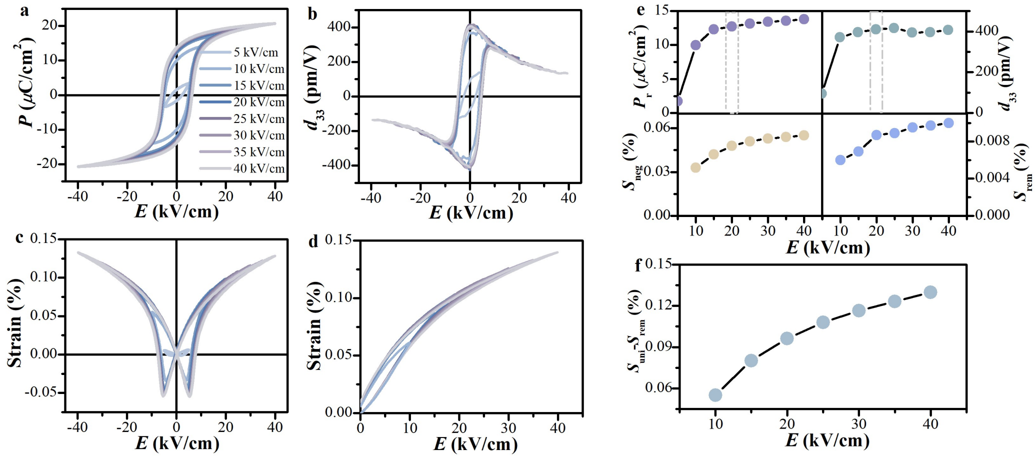

Supplementary Fig. 14 Ferroelectric, piezoelectric, and strain property as a function of electric fields. a *P-E* loop. **b** Electric field induced d_{33} curves. **c** Bipolar strain curves. **d** Unipolar strain curves. **e** P_r, d_{33}, S_{neg} and S_{rem}. **f** S_{uni}-S_{rem} under increasing electric field for x=0.045, measured at room temperature, 2nd cycle, and 1 Hz.

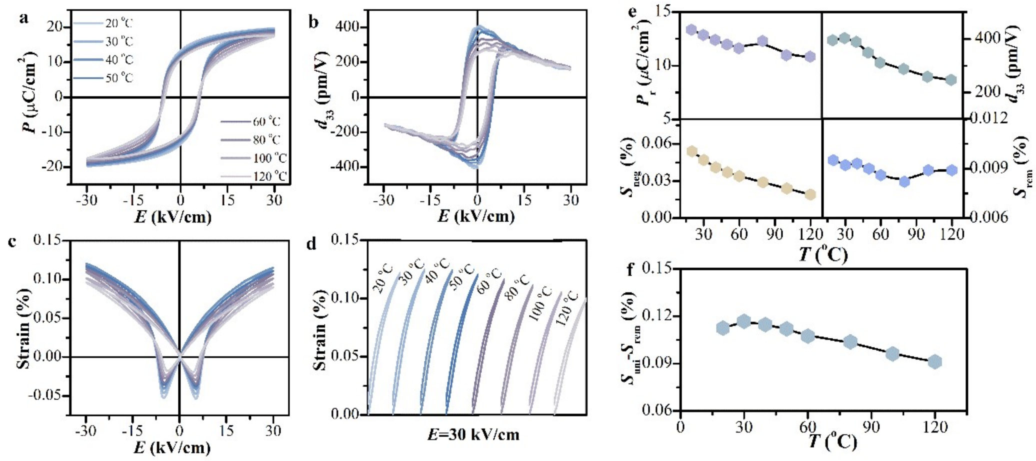

Supplementary Fig. 15. Ferroelectric, piezoelectric, and strain property as a function of temperature. a *P-E* loop. **b** Electric field induced d_{33} curves. **c** Bipolar strain curves. **d** Unipolar strain curves, **e** P_r, d_{33}, S_{neg} and S_{rem}. **f** S_{uni}-S_{rem} with increasing temperature for x=0.045, measured at 30 kV/cm, 2nd cycle, and 1 Hz.

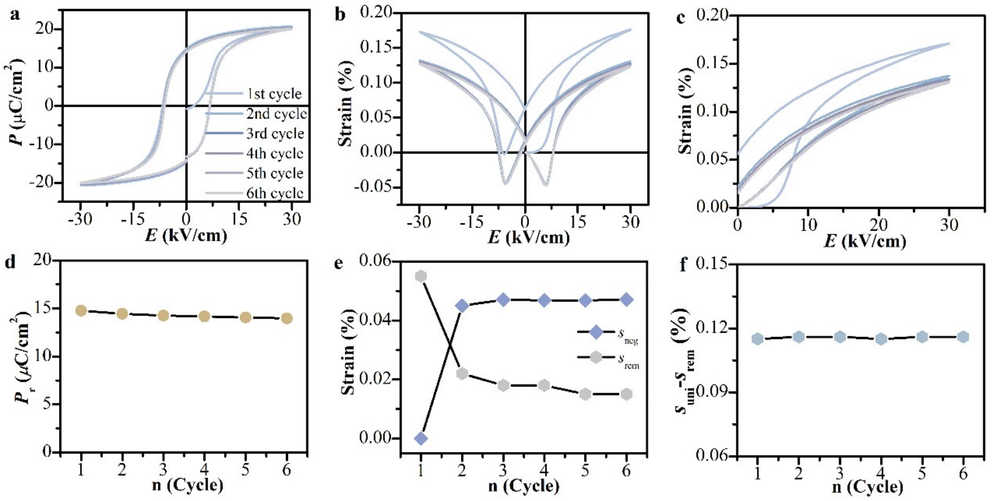

Supplementary Fig. 16 Ferroelectric and strain property as a function of cycle. a *P*-*E* loop. **b** Bipolar strain curves. **c** Unipolar strain curves, **d** P_r. **e** S_{neg} and S_{rem}. **f** S_{uni}-S_{rem} with increasing cycle for *x*=0.045, measured at 30 kV/cm, room temperature, and 1 Hz.

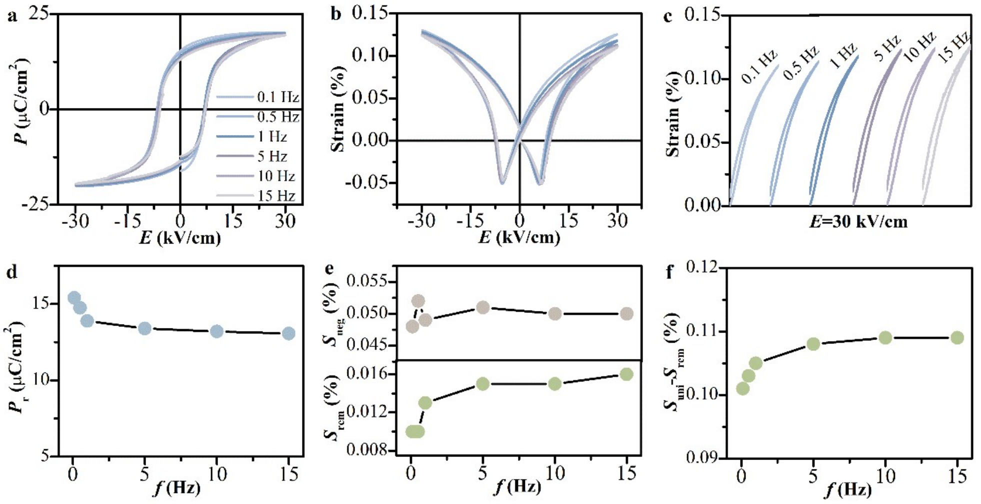

Supplementary Fig. 17. Ferroelectric and strain property as a function of frequency. a *P*-*E* loop. **b** Bipolar strain curves. **c** Unipolar strain curves, **d** P_r. **e** S_{neg} and S_{rem}. **f** S_{uni}-S_{rem} with increasing frequency for *x*=0.045, measured at 30 kV/cm, room temperature, and 2nd cycle.
